# Cardiovascular responses to orthostasis during a simulated 3-day heatwave

**DOI:** 10.1038/s41598-022-24216-3

**Published:** 2022-11-21

**Authors:** Jason T. Fisher, Urša Ciuha, Leonidas G. Ioannou, Lydia L. Simpson, Carmen Possnig, Justin Lawley, Igor B. Mekjavic

**Affiliations:** 1 Jozef Stefan International Postgraduate School, Ljubljana, Slovenia; 2 Department of Automation, Biocybernetics and Robotics, Jozef Stefan Institute, Jamova Cesta 39, SI-1000 Ljubljana, Slovenia; 3 Division of Performance Physiology and Prevention, Department of Sports Science, University of Innsbruck, Innsbruck, Austria; 4 Institute of Mountain Emergency Medicine, Eurac Research, Bolzano, Italy

**Keywords:** Blood flow, Vasodilation

## Abstract

Global warming has caused an increase in the frequency, duration, and intensity of summer heatwaves (HWs). Prolonged exposure to hot environments and orthostasis may cause conflicting demands of thermoregulation and blood pressure regulation on the vasomotor system, potentially contributing to cardiovascular complications and occupational heat strain. This study assessed cardiovascular and skin blood flow (SkBF) responses to orthostasis before, during and after a 3-day simulated HW. Seven male participants maintained a standard work/rest schedule for nine consecutive days split into three 3-day parts; thermoneutral pre-HW (25.4 °C), simulated HW (35.4 °C), thermoneutral post-HW. Gastrointestinal (T_gi_) and skin (T_sk_) temperatures, cardiovascular responses, and SkBF were monitored during 10-min supine and 10-min 60° head-up tilt (HUT). SkBF, indexed using proximal–distal skin temperature gradient (∆Tsk_P-D_), was validated using Laser-Doppler Flowmetry (LDF). The HW significantly increased heart rate, cardiac output and SkBF of the leg in supine; HUT increased SkBF of the arm and leg, and significantly affected all cardiovascular variables besides cardiac output. Significant regional differences in SkBF presented between the arm and leg in all conditions; the arm displaying vasodilation throughout, while the leg vasoconstricted in non-HW before shifting to vasodilation in the HW. Additionally, ∆Tsk_P-D_ strongly correlated with LDF (r = −.78, *p* < 0.001). Prolonged HW exposure and orthostasis, individually, elicited significant changes in cardiovascular and SkBF variables. Additionally, varying regional blood flow responses were observed, suggesting the upper and lower vasculature receives differing vasomotor control. Combined cardiovascular alterations and shifts towards vasodilation indicate an increased challenge to industrial workers during HWs.

## Introduction

Due, in part, to the ever-increasing concentration of greenhouse gases in the twentieth and twenty-first century, global temperatures have continuously risen, increasing the risk to health and wellbeing for billions of people worldwide. As global temperatures continue to increase, heatwaves (HWs) have become a regular occurrence; defined as a minimum of three consecutive days where the daily mean temperature exceeds 95% of the seasonal average^1^. The occurrence of these HWs has increased, as has their duration, and intensity. HWs can cause serious health consequences and risk of fatality to those experiencing them, due to the prolonged and excessive strain on the human body’s cardiovascular and thermoregulatory systems. Exceptionally severe HWs, such as the 2003 pan-European HW, can be responsible for tens of thousands of deaths; particularly in the elderly and vulnerable populations^2^.

The effects of HWs on humans can range from thermal discomfort and decrements in cognitive performance during complex tasks^3,4^, to more serious complications such as heat exhaustion, stroke, cramp, risk of syncope, and fatality^5–7^. Complications may, in part, be the result of excessive demand on the cardiovascular system, which needs to maintain both blood pressure and heat exchange^8^. These health issues may also vary due to a multitude of factors, including age, acclimatization, physical fitness, medical conditions, and environmental stress experienced^9^. Additionally, those working in a HW will likely experience orthostatic stress, the combination of which with heat stress is likely to be detrimental. During sustained heat stress and orthostatic challenge, ventricular filling and stroke volume are reduced due to blood pooling. This leads to sympathetically mediated adjustments for increases in heart rate (HR), cardiac contractibility and vascular resistance^10^. Lind, et al.^11^ reported a decrease in forearm blood flow whilst HR increased, however, these responses precede syncope, which is characterised by a significant fall in HR concomitant with a significant decrease in blood pressure. However, individuals can aid their thermoregulatory systems via changes in behaviour. These include seeking shelter, using cooling items such as water or fans, or wearing light-coloured clothing to reflect direct sunlight; the primary aim of which is the reduction of metabolic rate, consequently reducing the metabolic heat production^12^. Some occupations, however, limit individuals from behaviourally assisting thermoregulation. Occupational heat strain in jobs that require intense physical activity, protective clothing, and exposure to extreme ambient temperatures, such as the military, construction, agriculture, and tourism, are well documented^12–14^. Indoor workers though, who are also unable to control their external environment, have received far less scrutiny. Ciuha, et al.^3^ observed the impact of elevated ambient workplace temperatures, during a summer HW, which produced significant drops in manufacturing productivity (Odelo Slovenija d.o.o, Prebold, Slovenia). A systematic review by Flouris, et al.^15^ suggested that an estimated 35% of individuals working in hot conditions experience occupational heat strain, and 30% of workers report productivity losses. Understanding the cardiovascular and thermoregulatory challenges associated with indoor workers during a HW is essential for developing appropriate detection, prevention, and treatment techniques for workers.

The principal aim of the present study was to assess the cardiovascular and regional blood flow responses to HW conditions using a novel protocol: confining participants to laboratory-controlled ambient conditions for nine consecutive days, during which the first and last three days were normal temperature days, whereas days four to six simulated heat wave conditions. It was hypothesized that during a simulated 3-day HW:

1. Both gastrointestinal (T_gi_) and skin (T_sk_) temperatures would significantly increase, and thus indicate the onset of heat strain.
2. High ambient temperature conditions will result in increased peripheral perfusion augmenting heat loss.
3. Orthostatic stress will cause a reduction in blood flow to maintain arterial blood pressure.
4. The interaction of heat and orthostatic stress will produce regional differences (i.e., arms and legs) in the regulation of blood flow balancing the need to maintain arterial pressure and heat loss.

## Methods and materials

The present study was part of a larger project (European Commission Horizon 2020 project “Heat Shield”) investigating the effect of HWs on the health, well-being, and labour productivity of workers in industry. The aim was to simulate a schedule of work (0900–1800 h) and rest (1800–0900 h), as would be experienced by workers in the manufacturing industry, but under controlled conditions. Participants were confined to three areas (living quarters, work area, and cafeteria) at the Olympic Sport Centre Planica (Rateče, Slovenia).

### Experimental design

The study investigated the effect of a laboratory-controlled 3-day HW and orthostatic stress, on cardiovascular measures of heart rate (HR, b·min^-1^), stroke volume (SV, mL), cardiac output (CO L·min^-1^), systolic (SYS) and diastolic (DIA) blood pressure (mmHg), total peripheral resistance (TPR, mmHg·min·L^-1^), and skin blood flow (SkBF) reflected by proximal–distal skin temperature gradient (∆Tsk_P-D_). Thermal status was assessed by gastrointestinal (T_gi_) and skin (T_sk_) temperatures. Both cardiovascular and thermal variables were collected during a 10-min supine rest, and 10-min 60° head-up tilt (HUT) designed to introduce orthostatic stress. The 9-day experimental period commenced with a 3-day pre-HW period at neutral temperature (work: 25.4 ± 0.3 °C; rest: 22.3 ± 0.5 °C). A 3-day HW followed during which the diurnal and nocturnal ambient temperature increased in work and living areas (work: 35.5 ± 0.3 °C; rest: 26.3 ± 0.8 °C). The study concluded with a 3-day post-HW period, again at neutral temperature conditions. Ambient temperatures in the work areas were changed at 0700 h of days 4 and 7, so the participants arrived in the work area at the correct temperature. Similarly, the living areas were changed at 1200 h on days 4 and 7, so the participants returned to their living areas at the correct temperature. Relative humidity remained constant at 45% throughout the study in all areas, which is more commonly associated with dry HWs such as those observed in Europe and the Western US^16^. To avoid habitual acclimatization prior to the study, experiments took place in ambient conditions of 19.8 ± 1.8 °C Wet-Bulb Globe Temperature (from www.wunderground.com; accessed on 28 July 2021).

The ambient conditions throughout the whole study were simulated using data from a 3-day HW of similar intensity in Slovenia^3^. The 24-h ambient temperature within the facility did not mimic the sinusoidal change in ambient temperature observed in the mentioned HW but maintained the diurnal and nocturnal temperatures in the work and rest areas at average temperatures. Each day during working hours, the participants conducted low-intensity exercise sessions, comprising a stepping test (metabolic requirement of approximately 2.8 METS) in two 40-min sessions, and simulated work tasks (metabolic requirement of approximately 1.5 METS) for 2 h each day to represent a normal working day in industry; alongside cognitive testing. During the HW, participants were allowed a 1-h lunchbreak in normothermic conditions (25 °C), similar to industrial workers taking a break in a cooled canteen. The participants wore identical work overalls throughout the work period. The full global protocol used in the present study has been described previously Ioannou, et al.^17^

### Participant information

A sample size of seven participants was deemed to provide sufficient power to detect a statistical significance, assuming an α of 0.001 and β of 0.99 (G*Power Version 3.1.9.6, Germany). These results were calculated from the results of a previous study^18^ which described a 0.8% increase in labour loss for every degree increase in air temperature (R^2^ = 0.47). Ioannou, et al.^17^ further discuss the sample size calculation used in the present study. Thus, seven healthy young male participants were recruited to take part in the study; age: 21.5 ± 1.2 years; height: 180.0 ± 5.6 cm; body mass: 81.5 ± 14.5 kg; body mass index: 25.1 ± 4.0 kg.m^2^; fat mass: 22.5 ± 7.9%. All were non-smokers, engaged in regular physical activity recreationally, and were free from known cardiovascular, respiratory, and autonomic disease. The protocol was approved by the National Committee for Medical Ethics at the Ministry of Health of the Republic of Slovenia (approval no. 0120–402/2020/4: October 20th, 2020) and conformed to the guidelines of the Declaration of Helsinki, except for registration in a database. Prior to the start of the study, participants were familiarized with the study protocol and procedures, and gave their written consent for participation. Participants refrained from heavy exercise, alcohol, and caffeine throughout the entirety of the study. The participants’ diet was controlled and closely monitored throughout the study (Open Platform for Clinical Nutrition, www.opkp.si); set menus ensured the participants ate identical 3-day meal plans during each ambient condition.

Seven additional male participants (age: 27.7 ± 6.4 years; height: 178.7 ± 7.7 cm; body mass: 79.1 ± 9.2 kg; body mass index: 24.7 ± 2.0 kg.m^2^) were recruited for the validation of the ∆Tsk_P-D_ measurement, as an index of SkBF, against Laser Doppler Flowmetry.

### Experimental 60° head-up tilt (HUT) protocol

At the same time each day during the work period, participants underwent a 60° HUT test protocol. This was done in order to expose participants to stressors (ambient temperature, orthostatic stress) that would manipulate the baroreflex and thermoregulatory systems and produce a conflict in skin blood flow regulation. The HUT protocol was conducted 1.5-h after the first set of stepping exercise and simulated work task, and before the second set. All tests were conducted between 1300 and 1630 h, the order of participants remained the same on each day. The tests occurred at least 45 min after eating, not including time for instrumentation. Upon arrival, participants were instructed to lie in the supine position on the tilt table with their feet resting on a footplate. Following instrumentation, participants rested quietly for a period of 10 min to allow stabilization of all measured variables and to ensure an appropriate fluid stabilization of total body water^19^. Following this baseline period, participants were passively tilted to a 60° HUT position, where they remained for a further 10 min. While the test protocol would have been terminated early at the participants’ request or signs of presyncope based on ECG and beat-by-beat blood pressure recordings^20^, none of the participants requested premature cessation during HUT or showed haemodynamic signs of presyncope.

### Measurements

Cardiovascular parameters: Heart rate (HR) was determined from Lead II electrocardiogram. Arterial pressure was measured, in duplicate, via electro-sphygmomanometry (Tango, SunTechMedical Instruments Inc., USA) with a microphone placed over the brachial artery at the heart level to detect Korotkoff sounds, while the arm was parallel to the body. Beat-by-beat blood pressure was recorded via the volume clamp method (Finapres Nova; Finapres Medical Systems BV, Amsterdam, The Netherlands) and used to estimate beat-by-beat changes in stoke volume (SV, mL) and cardiac output (CO, L.min^-1^) using the Model Flow algorithm. Finometer values were calibrated against the average electro-sphygmomanometry brachial artery blood pressure measurements and corrected for the height of the participant. Total peripheral resistance (TPR) was estimated as the ration of mean arterial pressure (MAP) to CO.

Hydration levels were monitored daily with dipstick measurements, which measured urine specific gravity (USG) using the colorimetric method. Finger capillary (15 µL) blood samples were taken prior to the onset of the simulated HW, and upon its conclusion. All blood samples were analysed using an ABL80 FLEX CO-OX Blood gas analyser (Radiometer Medical, Brønshøj, Denmark), measuring haematocrit (Hct) and haemoglobin concentration (Hbc).

Deep body temperatures: The temperature of the gastrointestinal tract (T_gi_) was continuously measured using ingestible telemetric pills (Bodycap, Caen, France). These were ingested each morning upon being woken at a standardized time of 0700 h; measurement started at 0900 h. This allows appropriate time for the ingested pill to reach the stomach. For the purposes of the present study, deep body temperature measurements during the HUT protocol only were utilised.

Skin temperatures: The temperature of the skin (T_sk_) was measured at four sites continuously throughout each day, using thermistors (iButtons type DS1921H, Maxim/Dallas Semiconductor Corp. USA). For the purposes of the present study, skin temperature measurements during the HUT protocol only were utilised. Measurement sites were located at the chest, bicep, thigh, and calf; all on the right side of the body. An average skin temperature was calculated (Ramanathan, 1964):

1 $$T_{sk} = 0.3\left( {T_{chest} + T_{bicep} } \right) + 0.2\left( {T_{thigh} + T_{calf } } \right)$$

Proximal–distal temperature gradient (∆Tsk_P-D_): ∆Tsk_P-D_, an index of localised SkBF, was measured at 1-min intervals, between the forearm and fingertip (ΔTsk_forearm-fingertip_), and the calf and toe (ΔTsk_calf-toe_) (MSR145, MSR Electronics GmbH, Switzerland) on the left side of the body; areas considered to be heavily influenced by thermoregulatory and baroreflex mechanisms. Wireless thermistors (iButtons type DS1921H, Maxim/Dallas Semiconductor Corp. USA) at the forearm and fingertip also allowed continuous measurement of arm SkBF throughout each day. ∆Tsk_P-D_ was validated as a method of measuring peripheral SkBF against venous occlusion plethysmography in thermoneutral conditions (r^2^ = 0.98)^21^. Validation with Laser Doppler Flowmetry (LDF) also displays a strong correlation under anaesthesia (r^2^ = 0.63)^22^, and during steady-state exercise (r^2^ = 0.68)^23^. House and Tipton^24^ also suggested it is a suitable index, and recommended an adjusted scale, which was used in the present study (vasoconstriction: ≥ 2 °C; vasodilation: ≤ 0 °C). Since this indirect method of monitoring peripheral SkBF during an orthostatic tolerance test in normal and hot temperature conditions has not been validated previously, a separate validation was conducted to confirm ∆Tsk_P-D_ as an index of SkBF in the fingers and toes.

### Validation of ∆Tsk_P-D_

∆Tsk_P-D_ was validated as an index of SkBF by comparing the measurements with LDF (MoorVMS-LDF, Moors instruments, UK) in both the arm and leg. ∆Tsk_P-D_ and LDF were measured at the fingertip and great toe during supine and 60° HUT position, while exposed to ambient temperature of 25 °C and 35 °C in a climatic chamber. Immediately upon entry the participants entered the supine position, which was measured for 10 min, following which they were tilted into the 60° HUT position. The protocol was identical to the HUT protocol used in the 9-day HW simulation study. Spearman’s Rank correlation was calculated for the absolute measurements of ∆Tsk_P-D_ and LDF recorded during the final 2-min in each condition; considered a stable period.

### Data analysis

Cardiovascular data were collected on a beat-by-beat basis over the course of the 10-min supine and 10-min HUT postures; the final 2 min of each condition used as a stable period. T_gi_ and T_sk_ data were measured every minute for 22 h (T_gi_: 0900-0700 h; T_sk_: 2300-2100 h). T_gi_ and T_sk_ data were processed to produce minute averages over the course of the 10-min BL and 10-min HUT, of which the final 2-min of each posture were averaged. Minute averages of ΔTsk_forearm-fingertip_ and ΔTsk_calf-toe_ were collected for the duration of BL and HUT. Averages of the first two 3-day ambient conditions were calculated (non-HW and HW). Analysis of drawn blood via a radiometer provided values of haemoglobin (g·100 mL^-1^) and haematocrit (%). These values were used to calculate plasma volumes^25^ before and after the HW, thus providing an indication of any HW-induced change (ΔPV).

Two-way repeated-measures ANOVAs were used to assess the effects of two independent variables (ambient temperature, orthostasis) on the measurements recorded (HR, SYS, DIA, SV, CO, TPR, T_gi_, T_sk_, ΔTsk_P-D_). Simple main effects of these comparisons were also conducted. Partial Eta Squared was used to define the effect size in ANOVA tests. In each ANOVA, Mauchly’s Test of Sphericity was run for relevant variables, and the assumption of sphericity was met for each interaction if *p* > 0.05. Paired samples t-tests assessed the differences in the ∆Tsk_P-D_ responses of the arm and leg, in each of the four conditions produced by ambient temperature and posture (non-HW supine, non-HW HUT, HW supine, HW HUT); followed by Cohen’s D effect sizes. All statistical tests were completed using an alpha value of *p* < 0.05, with IBM SPSS Statistics (Version 26, IL, USA). Finally, Paired T Tests and Pearson Correlation Coefficient Tests were carried out to determine the significance of difference and relationship that exists between all pre-HW to post-HW measures, indicative of a HW related residual strain or adaptation.

### Institutional review board statement

The study was conducted in accordance with the Declaration of Helsinki, and approved by the Ethics Committee of Committee for Medical Ethics at the Ministry of Health (Republic of Slovenia) (Approval no. 0120–402/2020/4: October 20th, 2020).

### Ethics approval

National Committee for Medical Ethics at the Ministry of Health of the Republic of Slovenia (approval no. 0120–402/2020/4: October 20th, 2020).

### Consent to participate

All participants provided written informed consent for participation.

### Informed consent

Informed consent was obtained from all subjects involved in the study.

## Results

All participants successfully completed the 9-day confinement study. As reported previously^17^, all experienced a varying degree of thermal discomfort, considerable physiological strain and drop in productivity during simulated work and exercise tasks. There was no significant effect of HW on hydration status as reflected in the USG measured during the non-HW (1.0239 ± 0.0043) and HW conditions (1.0224 ± 0.0036) (*p* = 0.156), suggesting appropriate maintenance of euhydration.

### Deep body and skin temperatures

In the non-HW, T_sk_ stabilised at 33.5 ± 0.5 °C, whilst in the HW ambient conditions T_sk_ increased to 35.9 ± 0.3 °C. Similarly, there was an increase in the deep body temperature from the non-HW (37.2 ± 0.2 °C) to the HW ambient condition (37.4 ± 0.2 °C), though to a lesser extent. Thus, between the non-HW and HW, there was a significant increase in T_sk_ (t = 24.06, *p* < 0.001), and also in the T_gi_ (t = 2.368, *p* = 0.029). In addition, there was a significant effect of participants posture (supine, HUT) on T_sk_ (F = 12.494, *p* = 0.002, η_p_^2^ = 0.103), but no significant effect on T_gi_. Skin and deep body temperatures in each time period over the full 24-h are presented in Table 1.

**Table 1:** Measured mean (± SD) and range of deep body and skin temperatures over the 24-h period during work, rest, and sleep periods. Temperature is presented as a mean of both 3-day non-HW and HW conditions.

	Deep Body temperature (°C)		Mean Skin temperature (°C)	
	Mean (± SD)	Range (Min–Max)	Mean (± SD)	Range (Min–Max)
**Work period**				
Pre-HW	37.1 (0.3)	36.4–37.8	33.3 (0.4)	31.8–34.3
HW	37.3 (0.3)	36.6–38.0	35.7 (0.3)	34.0–36.3
**Rest period**				
Pre-HW	37.3 (0.1)	37.0–37.6	33.2 (0.5)	31.9–34.1
HW	37.5 (0.1)	37.2–37.8	34.2 (0.6)	33.0–35.8
**Sleep period**				
Pre-HW	36.5 (0.3)	36.2–37.7	34.1 (0.4)	32.3–34.8
HW	36.6 (0.4)	36.1–37.7	34.3 (0.3)	33.7–34.9

Work period: 09.00–18.00. Rest period: 18.00–23.00. Sleep period: 23.00–07.00. Pre-HW: Average of testing days 1–3. HW: Average of testing days 4–6. Ambient temperature.

### Cardiovascular measures (HR, SYS, DIA, SV, CO, TPR)

**Figure 1:**
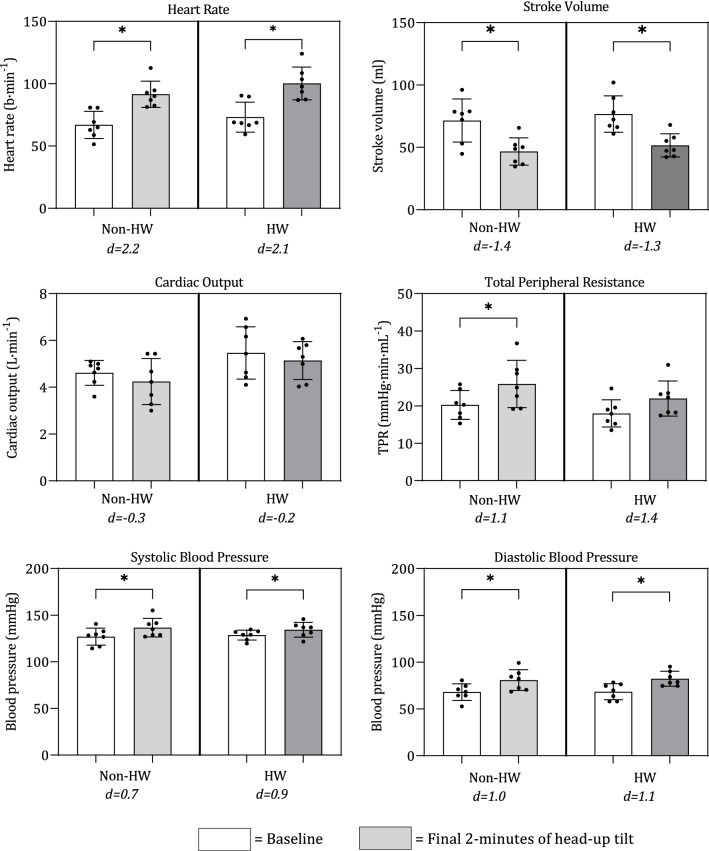
Cardiovascular responses to 60° head-up tilt (HUT) during non-HW and HW. Mean ± SD and individual responses to heart rate (HR, b·min^-1^), stroke volume (SV, ml), cardiac output (CO, L·min^-1^), mean arterial pressure (MAP, mm Hg), and total peripheral resistance (TPR, mmHg·L·min^-1^). Significant differences between postures in each condition denoted with an asterisk (*). Cohen’s D effect sizes presented below ambient conditions, indicating the size of effect between supine and HUT in each ambient condition.

The combined effects of changes in ambient temperature and posture did not produce a significant interaction effect of any of the cardiovascular measures. The simple main effects, however, did produce significant differences in certain cardiovascular variables, as described below. Figure 1 displays the cardiovascular responses during supine and HUT, in both the non-HW and HW; significant differences between postures are denoted with an asterisk (*). Plasma volume did not differ significantly between the non-HW and HW, with an average ΔPV of − 0.03% (*d* = − 0.1).

The HW condition caused a significant increase in both HR (F = 9.104, *p* = 0.008, η_p_^2^ = 0.349) and CO (F = 5.246, *p* = 0.035, η_p_^2^ = 0.236). HR increased from a mean non-HW value of 67.1 ± 11.2 to 73.1 ± 12.2 b·min^-1^ during the HW, whilst CO increased from a non-HW value of 4.6 ± 0.9 to 5.5 ± 1.8 L·min^-1^ during the HW. Notably, TPR decreased from non-HW value of 20.2 ± 5.5 to 17.9 ± 7.9 mmHg·L·min^-1^ during the HW with a medium to strong effect size (η_p_^2^ = 0.127)^25^, however, this change was deemed to be non-significant.

Orthostatic stress, induced by HUT, caused significant increases in HR (F = 130.137, *p* < 0.001, η_p_^2^ = 0.884), SYS (F = 13.167, *p* = 0.002, η_p_^2^ = 0.927), DIA (F = 24.419, *p* < 0.001, η_p_^2^ = 0.996), and TPR (F = 7.874, *p* = 0.012, η_p_^2^ = 0.317). Conversely, SV decreased significantly (F = 43.717, *p* < 0.001, η_p_^2^ = 0.720), whilst CO displayed no significant change during HUT. HR increased from 70.2 ± 1 to 96.2 ± 13.3 b·min^-1^, SYS increased from 127.8 ± 11.5 to 135.8 ± 11.8 mmHg, DIA increased from 68.1 ± 11.7 to 81.7 ± 11.2 mmHg, and TPR increased from 19.0 ± 6.9 to 23.9 ± 8.0 mmHg·L·min^-1^. For SV, the effect of HUT caused a decrease from 74.2 ± 21.5 to 49.3 ± 14.3 mL.

### Regional proximal–distal skin temperature gradient (index of SkBF)

**Figure 2:**
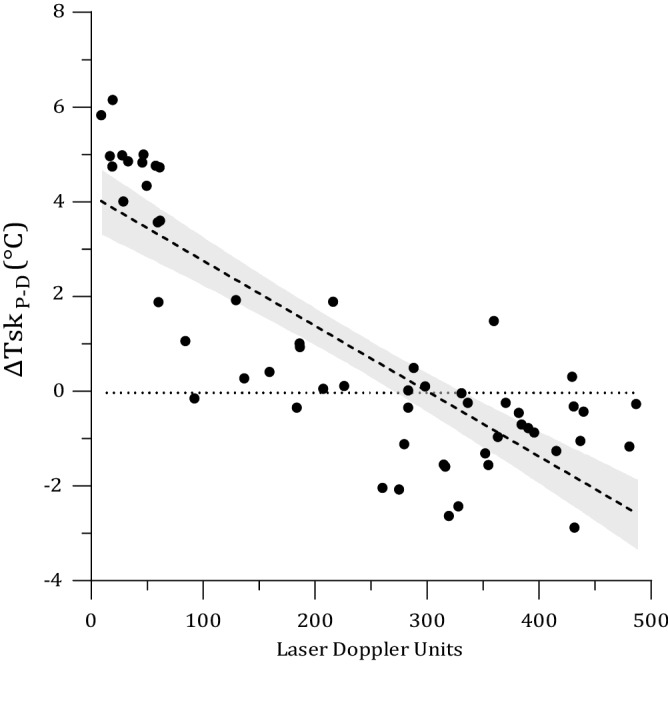
Relationship between ∆TskP-D and LDF measurements, with associated regression line. Data points relate to measurements recorded during the final 2-min in each condition (supine or HUT).

The validation of ∆Tsk_P-D_ as an index of SkBF revealed a significant negative correlation (Fig. 2) between ∆Tsk_P-D_ and LDF measurements (r = − 0.776, *p* < 0.001). The measurements were correlated using absolute values (LDF: Laser doppler units; ∆Tsk_P-D_: °C); median and range values were also calculated for LDF (median: 277.3; range: 477.8) and ∆Tsk_P-D_ (median: 0.0; range: 9.0).

**Figure 3:**
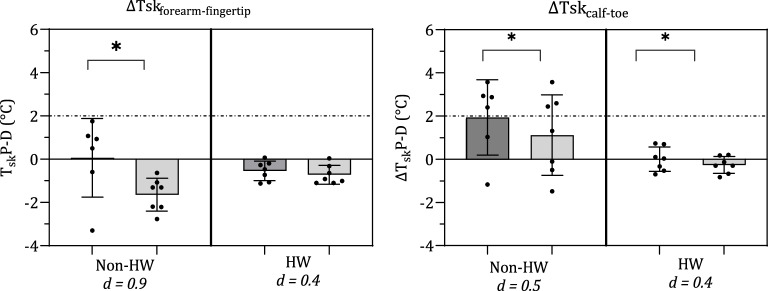
Mean ± SD and individual responses of skin blood flow responses (ΔTsk_forearm-fingertip_, ΔTsk_calf-toe_) to 60° head-up tilt during the non-HW and HW. Significant differences between postures in each condition denoted with an asterisk (*). Dotted line indicates threshold for onset of vasodilation, solid line indicates threshold for onset of vasoconstriction. Cohen’s D effect sizes presented below ambient conditions, indicating the size of effect between supine and HUT in each ambient condition.

The interaction of change in ambient temperature (non-HW, HW) and posture (supine, HUT) revealed a significant combined effect on ∆Tsk_P-D_ in the arm (F = 8.069, *p* = 0.014, η_p_^2^ = 0.383) but not in the leg. Figure 3 displays the ∆Tsk_P-D_ responses of the arm and leg during supine and HUT, in both the non-HW and HW.

The simple main effect of change in ambient temperature caused a significant decrease in the ΔTsk_calf-toe_, decreasing from 2.2 ± 0.9 °C in the non-HW to 0.0 ± 0.6 °C in the HW (F = 9.174, *p* = 0.010, η_p_^2^ = 0.414); indicating a shift towards vasodilation. The ΔTsk_forearm-fingertip_, despite crossing the suggested threshold for vasodilation^24^ from 0.0 ± 0.9 to − 0.6 ± 0.4 °C, did not produce a significant response to ambient temperature.

In response to HUT, the second simple main effect, both the ΔTsk_forearm-fingertip_ and ΔTsk_calf-toe_ displayed a significant decrease, indicating a shift towards vasodilation. The ΔTsk_forearm-fingertip_ decreased from − 0.3 ± 0.9 to − 1.0 ± 0.8 °C (F = 5.918, *p* = 0.030, η_p_^2^ = 0.313), whilst the ΔTsk_calf-toe_ decreased from 1.0 ± 1.6 to 0.3 ± 1.4 °C (F = 9.009, *p* = 0.010, η_p_^2^ = 0.409).

As described above, differences lay in the blood flow of the arms and legs, as indexed by the ∆Tsk_P-D_. When comparing between the arm and leg response in each condition, significant differences lay in each of the four conditions as produced by combining ambient conditions and postural changes: non-HW supine (t = − 5.001, *p* < 0.001, *d* = 1.1), non-HW HUT (t = − 4.934, *p* < 0.001, *d* = 1.2), HW supine (t = − 3.822, *p* = 0.001, *d* = 0.9), HW HUT (t = − 2.244, *p* = 0.036, *d* = 0.6).

### Adaptation to the simulated HW

**Figure 4:**
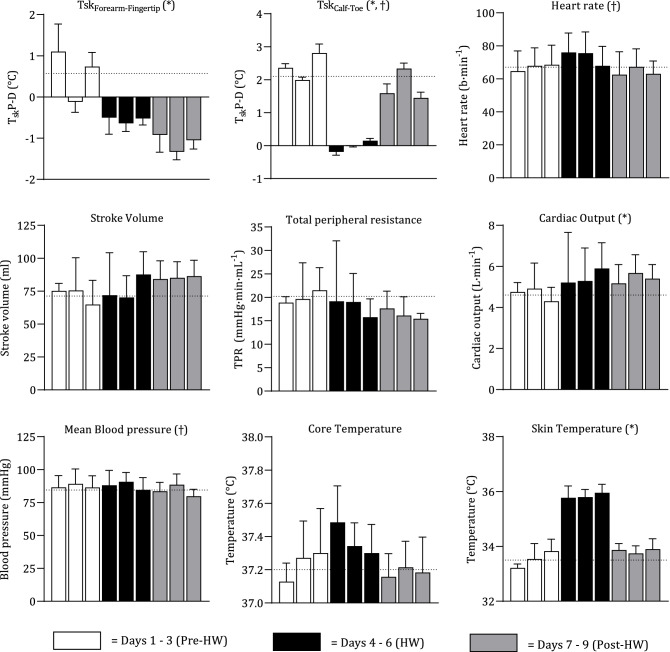
Mean ± SD of skin blood flow and cardiovascular responses in baseline position (supine) on each individual day. White bars indicate pre-HW (days 1–3), black bars indicate HW (days 4–6), and grey bars indicate post-HW (days 7–9). Dotted line displays the average value of the pre-HW period. † indicates a significant difference between pre-HW and post-HW, * indicates a significant correlation between pre-HW and post-HW.

Pre-HW to post-HW significant differences were identified in ΔTsk_forearm-fingertip_ (t = 13.40, *p* < 0.001), ΔTsk_calf-toe_ (t = 6.017, *p* < 0.001), CO (t = 2.739, *p* = 0.034), and T_sk_ (t = 3.418, *p* = 0.014). Significant pre-HW to post-HW correlations were only observed in ΔTsk_calf-toe_ (r = − 0.5685, *p* = 0.04), and in mean blood pressure (MBP; r = 0.7633, *p* = 0.023). Figure 4 displays the mean (± SD) responses of all variables on individual days, measured in the baseline (supine) position.

## Discussion

The present study utilized a novel method of confining participants to simulated HW conditions, to assess the cardiovascular strain experienced by those working in industry during a HW. By simulating ambient conditions measured in an industrial environment during a HW in Slovenia^3^, an understanding of the effects of a HW could be measured in controlled conditions. Perhaps unsurprisingly, the effect of increased ambient temperatures caused a significant increase in both T_gi_ and T_sk_, confirming hypothesis one. As a result, the main findings of the study were that prolonged exposure to HW conditions and orthostasis, independently, significantly affected cardiovascular and SkBF responses; thus, confirming hypotheses two and three. Indeed, while the interaction of higher ambient heating and orthostatic stress produced no significant effect on cardiovascular variables, a significant regional effect on SkBF was observed. The results, and the physiological mechanisms that drive them, indicate a higher propensity for heat strain in occupational workers during a HW.

### Deep body and skin temperatures

As expected, when exposed to elevated ambient temperatures, T_gi_ rose passively as heat production outweighed heat loss; though this was deemed to be a sustainable increase rather than an uncompensable gain. The T_sk_, which is much more responsive to external changes in temperature, varied considerably between ambient conditions. The changes observed are likely to reflect the T_gi_ measured, albeit not precisely due to large fluctuations that are seen during lunch breaks (12.00–13.00 h). However, it is worth nothing that the deep body and skin temperatures described in the present study relate specifically to the HUT protocol. For these reasons, fluctuations observed in the data do not affect the results described. Table 1, however, displays a greater core temperature in the rest conditions than the work conditions, which represent a lower ambient temperature. This trend is observed regardless of simulated-HW conditions and is likely the result of circadian rhythm causing a peak core temperature at ~ 2100 h^26^. Overall, the responses of the T_gi_ and T_sk_ reveal that a degree of thermal strain was experienced by the participants, which was suitable to elicit cardiovascular and skin blood flow responses.

### Cardiovascular Variables

High ambient temperatures can passively raise T_gi_ and T_sk_ to levels that initiate heat adaptation, causing several significant physiological improvements such as reduced resting T_gi_, enhanced sweat capacity, and lower resting/exercising heart rate^27^. Conversely, individuals experiencing HW conditions, without prior acclimatization, appear to suffer adverse physiological effects. On HW days, as expected, there was a significant increase in HR which enabled a raised CO to sustain a greater level of SkBF required for heat dissipation. These findings reflect those of Crandall, et al.^28^ who identified an affected vagal modulation of HR during heat stress, leading to a decrease in cardiac vagal activity whilst simultaneously increasing sympathetic activity; possibly contributing to orthostatic intolerance. The cardiovascular responses to orthostatic stress are well known, and the present study further consolidates these responses. Increases in HR, SYS, DIA, and TPR are the result of peripheral vasoconstriction in lower limbs, largely mediated by the baroreflex system and the venoarteriolar reflex. These responses are produced when an increase in arterial pressure is detected, reducing baroreceptor afferent discharge, and triggering reflex increases in HR, cardiac contractility, and vascular resistance to reduce blood pooling and promote venous return for vital organs^29^. Conversely, a decrease in SV was observed, occurring when venous pooling and capillary filtration reduce the circulating blood volume by 500–1000 mL^30^, the result of which is lower venous return and subsequent outgoing stroke volume. In the present study, CO was not significantly higher during orthostatic stress compared to the supine position. This is unusual, particularly considering the relatively young age of the participant sample. Work by Hainsworth and Al-Shamma^31^ assessing the cardiovascular response to orthostasis in different age groups also observed a decreasing cardiac response to orthostasis with age, which is concurrent with additional research^32^.

### Regional proximal–distal skin temperature gradient (index of blood flow)

The method of measuring SkBF in the present study was ∆Tsk_P-D_, a non-invasive and robust method. A validation test using seven different participants ensured that these measurements were valid and reliable in the ambient conditions and postures used in the present study, to aid those results already established by previous studies^21–23^. Each of these previous studies proposed varied correlation coefficients in differing conditions, ambient temperatures, and using different measurement techniques with which ΔTsk_P-D_ was compared against. None of these previous validations have considered the changes in blood flow that occur with changes in ambient temperature from 25 °C to 35 °C, changes in posture from supine to HUT, and in different regions of the body; simultaneously. The present study considered the interaction of all external variables on ∆Tsk_P-D_, allowing a more conclusive relationship between measurement techniques to be drawn. The evidence obtained provides support that ∆Tsk_P-D_ may be used as a suitable index of finger and toe SkBF in place of more expensive, less robust, and less invasive alternatives. Additionally, the results of this validation also displayed regional differences in the SkBF of individuals, when experiencing changes in posture and ambient temperature. These results further support the hypotheses outlined in the present study.

Both ambient temperature and posture (supine, HUT) independently appeared to significantly affect the ΔTsk_P-D_, however, their combined influence was only observed in the ∆Tsk_forearm-fingertip_. This agrees with Crossley, et al.^33^ who explains that during lower-body negative pressure (LBNP), vasoconstriction in the forearm occurs with whole-body heating, as a result of the baroreflex reducing the SkBF; implying that the baroreceptor reflex can override thermoregulation. Additionally, Lind, et al.^11^ suggest that during local or whole-body heating, the baroreceptor-mediated regulation of SkBF is still maintained. The effects of ambient temperature and orthostasis independently were also significant, and were also noted to differ regionally. The substantial regional differences in ∆Tsk_P-D_ occurred in both normothermia (non-HW) and HW conditions, with consistently higher cutaneous vasodilation observed in the hands than the feet. The results agree with previous speculation^34^ that the vasomotor response of the arms is driven predominantly by thermoregulatory systems, whereas the legs are influenced predominantly by baroreflex actions to maintain blood pressure, as indexed by vasodilation and vasoconstriction. The greater vasodilation observed in the fingers in all measured conditions potentially suggests a predominant influence of thermoregulatory drive, reflected in greater sensitivity to changes in temperature^35^ in the finger than in the toe. ∆Tsk_calf-toe_ in the non-HW was significantly higher indicating vasoconstriction of the cutaneous vasculature, and during the HW both regions demonstrated a decrease in ∆Tsk_P-D_, indicative of greater SkBF; suggesting an integrated reflex to promote vasodilation and withdraw vasoconstrictor drive, occurring as a ‘thermal threshold’ is met. This integrated regulation is described by Heistad et al.^36^ whereby the forearm, containing both cutaneous and muscular vasculature, displayed increased vasodilator reflex responses to heating. During the HW, the shift in blood flow to the skin may indicate an active vasodilator drive accounting for 80–90% of cutaneous vasodilation^8^. Though the present study cannot confirm this theory as sympathetic nerve activity was not measured.

Previous research also agrees with the regional differences observed when gravitational stress is applied. Stewart^37^ noted a stronger inverse relationship between blood pressure and SkBF in the leg than in the arm, implying a greater importance of leg circulation in blood pressure maintenance. Based on their findings, Kitano, et al.^38^ described complementary evidence, observing a greater vasoconstrictor response in the lower body during HUT. Possible reasons cited include differing sensitivity of sympathetic nerve activity between the arm and leg, and modulation of vascular responses via local mechanisms in the leg. Those working in industry during a HW are at direct risk of orthostatic intolerance, as venous pooling in the legs may cause syncope if integrated vascular balance is not achieved.

### Adaptation to the simulated-HW

While it is clear there is an acute cardiovascular response during a simulated-HW, it is also important to consider the prolonging and residual effects of said HW upon return to normothermic conditions. During the 2003 European HW, Pirard, et al.^39^ indicated that in France alone the daily number of excess deaths reached as high as 2197 on the final day of the heatwave. Upon return to non-HW temperatures, these daily numbers decreased day-by day due to, in part, a reduction in the cardiovascular strain. However, while a reduction is observed each day, it takes up to seven days for the number of deaths to return to normal levels; indicating a prolonged effect of the HW. This trend is also observed during other HWs, such as the 1995 Chicago HW^40^ and various English summer HWs^41^.

The results of the present study indicate a severe increase in the forearm skin blood flow post-HW, as indexed by Tsk_Forearm-Fingertip_, rising to levels higher than that of the HW. In addition, there is also an increase in leg blood flow during this time. These changes are enabled by a significantly higher CO observed, which consequently result in an increase in the T_sk_ in the post-HW when compared to the pre-HW. These results certainly indicate a prolonged effect of the HW, though it appears these effects are a positive adaptation occurring in the body to respond to heightened ambient temperatures. Results such as these are not dissimilar to those observed in active heat acclimation studies^42–45^, which observed an increase in SkBF at a given deep body temperature, indicating a reduction in the thermal threshold required for vasodilation. However, heat acclimation protocols normally incorporate high levels of exercise and repeat exposures to heat, to maintain an elevated core temperature^46–48^; which are inconsistent with the present study. Studies assessing thermal therapy over a longer duration have described improvements in brachial artery endothelial function^49,50^, similar to that of moderate-intensity exercise. Thus, adaptation may occur in place of negative cardiovascular responses, congruent with an increased risk of morbidity. The younger age and physical health of the participants in the present study may have exaggerated these results, and it is possible that elderly and more vulnerable populations, who are at greater risk from extreme HWs, may not exhibit the same positive adaptations^51,52^. It is therefore of importance that the future research studying this adaptation (or lack thereof) should seek to develop an understanding of the responses of elderly and vulnerable populations, to these increasingly regular yet adverse ambient conditions.

### Limitations

The median age of manufacturing workers is 44.5 years (available from: www.statista.com; accessed 2 September 2021), and females represent 46.2% of the labour force (available from: www.appsso.eurostat.ec.europa.eu; accessed on 11 August 2021). To fully appreciate the risk of a HW to those in industry, these populations should also be considered, as the young male sample group in the present study may not reflect the responses of the workforce in the manufacturing industry. A Heat Shield Technical report^53^ of workers during a HW details 37% and 47% of males experiencing headaches or exhaustion, respectively, compared to 73% and 64% of females, respectively; additionally, 33% of females experienced vomiting or nausea. In addition, during orthostasis, ΔTsk_forearm-fingertip_ and ΔTsk_calf-toe_ will have been influenced partially by the venoarteriolar reflex^54^. However, measurements of neural pathways were not conducted in the present study, so its influence is hard to quantify; future research should endeavour to understand the venoarteriolar reflex’s role in regional variation of SkBF.

### Conclusion

The present study used a protocol simulating HW conditions and application of an orthostatic stress to identify cardiovascular and thermoregulatory strain in workers. The novelty of the study design revealed that workers exposed to prolonged and high ambient temperatures, concomitant with orthostatic stress, does not produce significant changes in the cardiovascular responses, though individually these stressors have significant impacts. In addition, these combined influences produced significant changes in T_gi_, T_sk_, and SkBF; early indicators of heat stress. Additionally, the study further detected regional variations in SkBF, as represented by ΔTsk_P-D._ These responses represent the apparent cardiovascular strain experienced by industrial workers during HWs, which may lead to heat illness, syncope, or potential fatality.

## Data Availability

Data available upon request. Please contact Prof. Igor B. Mekjavic (email address: Igor.mekjavic@ijs.si).
